# Effects of diet supplemented with water extracts of *Artemisia annua* L. on small intestinal immune and antioxidative indexes in lambs

**DOI:** 10.3389/fvets.2025.1545729

**Published:** 2025-07-23

**Authors:** Gen Gang, Ruiheng Gao, Huricha Zhao, Xiao Jin, Yuanyuan Xing, Lei Hong, Sumei Yan, Yuanqing Xu, Binlin Shi

**Affiliations:** College of Animal Science, Inner Mongolia Agricultural University, Hohhot, China

**Keywords:** water extracts of *Artemisia annua* L, lamb, small intestine, immune index, antioxidative index

## Abstract

**Introduction:**

*Artemisia annua* L., an herbaceous plant, belong to the Artemisia genus within the Asteraceae family. Due to its significant medicinal properties, it has emerged as a focal point of research in the field of animal production. In the present study, the responses of intestinal immune and antioxidative indexes, and the related gene expression to water extracts of *Artemisia annua* L. (WEAA) supplementation in diet were profiled in lambs.

**Methods:**

In total, 32 female lambs (Dorper × Han), with eight replicates per group, were randomly assigned to four treatment groups. These groups were created by supplementing 0, 500, 1,000, and 1,500 mg/kg WEAA to the basal diet, respectively.

**Results:**

The results showed that WEAA addition increased sIgA, IgG, IL-1β, IL-2 and IL-4 levels in the duodenal and jejunal mucosa in a manner that was dependent on the dosage (*p* < 0.05). Moreover, WEAA promoted the expression of factors (*TLR4, MyD88, IKKβ, IκBα, NF-κB p50, NF-κB p65, IL-1β* and *IL-4*) related with the TLR4/NF-κB pathway, thus improving small intestinal immune function, thereby showing peak effects in the 1,000 mg/kg WEAA group. Additionally, WEAA supplementation also enhanced antioxidative function through the Nrf2/Keap1 pathway in the small intestinal mucosa, particularly by increasing GSH-Px and CAT concentrations and decreasing MDA content in a manner that was dependent on the dosage (*p* < 0.05), with maximal effects observed in the 1,000 mg/kg group. Furthermore, expressions levels of *Nrf2*, *GSH-Px* and *HO-1* in the small intestine increased quadratically (*p* < 0.05), while *Keap1* expression levels exhibited a downward quadratic trend (*p* < 0.10).

**Conclusion:**

In summary, the optimal dietary addition of 1,000 mg/kg WEAA significantly enhanced intestinal immune function, antioxidant capacity, and the expression of related genes in the intestinal mucosa of lambs.

## 1 Introduction

In recent decades, the global sheep industry has undergone a significant transformation from traditional extensive grazing systems to modern intensive production models. Due to increased demand for lamb and its by-products, land degradation problem is increasingly serious along with the pursuit of industry to improve the market competitiveness of livestock products and other issues (1, 2). In contemporary sheep production systems, this shift has brought about substantial changes to the natural habitat and innate behavior of sheep, which are likely to have negative effects on their metabolism, immune function and health (3, 4). These changes are different from the natural conditions to which sheep have been adapted for a long time (5). In addition, sheep health and productivity are heavily influenced by their diet composition, which holds a key position in growth performance, body development, and immune function, but the nutritional requirements of sheep are often easily overlooked in intensive production systems (6). Therefore, there has been a growing interest in improving diet nutrition strategies to enhance lamb health and performance. Among these strategies, natural plant-derived feed additives have potential to be a suitable alternative to synthetic growth promoters and alternatives to antibiotics in animal production in recent years (7–9).

The natural additives have been shown to have safe and reliable positive effects in livestock and poultry production, to have high consumer acceptance, and have the potential to promote animal growth and health without the developing resistance (10). *Artemisia annua* L. (*A. annua*), a member of the family Asteraceae which is considered native to Asia with its origin in China. *A. annua* is commonly known as sweet wormwood (11). This plant has a long history of being used as a traditional herbal remedy for malaria in China. Extensive research has been conducted on the *A. annua* and its extracts owing to their rich phytochemical profile and diverse biological activities, including polysaccharides, flavonoids, sesquiterpenes (artemisinin), phenols, essential oils, etc. (10, 12). Artemisinin is globally recognized for its effectiveness against malaria. In addition to its anti-malaria properties, *A. annua* also exhibits various other significant biological activities, including antibacterial, anti-inflammatory, anti-tumor, and antioxidant effects (13, 14).

In a series of studies involving different species, the multiple benefits of *A. annua* and its extracts on animal nutrition and health have been scientifically validated. Studies using rodents and poultry as models have shown that adding an appropriate amount of *A. annua* extract to the feed can significantly improve growth performance, optimize intestinal health, and enhance antioxidant status. Zhang et al. (15) evaluated immunomodulatory and antioxidant capabilities of polysaccharides derived from *A. annua*. These polysaccharides showed significant immunostimulatory activity and antioxidant effects, promoting the synthesis of TNF-*α* and IL-6 in mouse macrophages. Cui et al. (16) studied the effect of 1% *A. annua* supplemented to the diet on antioxidant capacity in serum and jejunum of geese. The results showed improved antioxidant enzyme activity (CAT, GSH-Px, SOD, T-AOC) and decreased MDA contents. Guo et al. (17) reported that the supplementing polysaccharides extracted from *A. annua* to the diet could significantly increase the expression of *CAT* and *Nrf2* in the small intestinal mucosa of broilers. This suggest that the enhancement of antioxidant enzyme activities may be through the Nrf2 signaling pathway. Similarly, in the field of aquaculture, *A. annua* leaf extract has been found to increased serum immunoglobulin levels, and activity of CAT and GSH-Px, while decreasing the content of malondialdehyde (MDA) in serum of Common Carp (18). In a study on weaned pigs, Niu et al. (19) found that the enzyme-treated *A. annua* (EA) affected the intestinal immune ability in weaned pigs, and the EA dosage of 2 g/kg decreased levels of IL-1β, IL-6, and TNF-αin the jejunum and increased the concentration of immunoglobulin (Ig) A and IgG in the ileum compared to the CON group. Song et al. (20) demonstrated that EA the intestinal inflammatory response of heat-stressed broilers, and found that EA could significantly increase IL-10 production and decrease *TLR4* mRNA expression in both the jejunum and ileum, suggesting that EA can inhibit TLR4/NF-κB pathway activation in heat-stressed conditions, thereby reducing inflammation. In addition, studies have shown that the regulatory mechanisms of Artemisia plants and their extracts are related to recognition of bio-active compounds by TLR4 on the surfaces of immune cells. This recognition triggers a series of signaling pathways, among which the NF-κB pathway is an extremely important pathway in cellular signaling and plays a key role in regulating the body’s immune inflammatory response (21, 22). The results indicate that *A. annua* may have a relatively positive effect in different livestock species, including sheep. The intestinal tissue serves as both a physical and immune barrier against pathogens and toxins, playing a crucial role in nutrient absorption and immune system (23). Enhancing intestinal immunity and antioxidant function through dietary interventions may improve lambs’ health and productivity.

The potential advantages of *A. annua* in animal nutrition have been demonstrated across several animal species. However, there is limited research exploring its role in sheep nutrition. Based on the unique physiological and nutritional demands of lambs, as well as the special challenges associated with intensive farming models, it has become increasingly important to study the effectiveness of *A. annua* supplements. Consequently, there is demand for further research into the impact of supplementing with WEAA on the intestinal immunity and antioxidant ability of lambs. These studies will provide valuable insights into the potential of *A. annua* as a natural feed additive in intensive sheep production, and may offer a sustainable and effective approach to improving lamb health and productivity. This research endeavor aims to fill this knowledge gap by evaluating the effects of WEAA supplementation on the intestinal immunity, antioxidant function, and their underlying molecular regulation in lambs, all within the context of conventional feeding practices.

## 2 Materials and methods

The animals used in the study were from a sheep farm in Hohhot, Inner Mongolia, China. All experimental protocols were approved by the Animal Care and Use Committee of Inner Mongolia Agricultural University (approval no. NND2021097) and followed the ethical guidelines for animal research established by the Laboratory Animal Sciences and Technical Committee (SAC/TC281) of the Standardization Administration of China, in accordance with *Laboratory animal—Guideline for ethical review of animal welfare* (GB/T 35892-2018) (24, 56).

### 2.1 Animals, diets, and design

In total, 32 female lambs (Dorper × Han) aged 3 months, weighing 24 ± 0.09 kg each, were recruited to the study. These lambs were divided into 4 groups, with 8 lambs in each group. The control group was fed solely a basal diet, whereas the treatment groups were provided with the same basal diet supplemented with different amounts of WEAA: 500, 1,000, and 1,500 mg/kg, respectively. The feeding trial lasted for 75 days (adaptation phase: 15d; formal experimental phase: 60d). The lambs were provided with pelleted feed twice a day (at 08:00 and 16:00 h) with *ad libitum* access to experimental diets and water. Each of the lambs was kept in individual pen, and their feed intake was carefully monitored to make sure that any leftover feed was over 5% of the total supply. The basic diet was designed to fulfill the Nutritional Requirements of Meat-type Sheep by NY/T 816–2021 (57). In Table 1, the feed components and the chemical makeup of the basal diet are outlined. Small intestinal (duodenum, jejunum and ileum) tissue samples were gathered to assess immune and antioxidative factors, along with the expression of related genes.

**Table 1:** The composition and nutrient levels of the basal diet (air-dry basis, %).

Ingredients	Content	Nutrients composition^b^	Level
Alfalfa hay	16.25	Digestible energy (MJ/kg)	12.01
Corn straw	14.00	Dry matter	89.89
Oat grass	24.75	Crude protein	15.80
Corn	23.25	Neutral detergent fiber	40.80
Soybean meal	10.95	Acid detergent fiber	26.24
Wheat bran	4.25	Calcium	1.08
Corn germ meal	1.95	Phosphorus	0.40
Soybean oil	1.10		
Premix^a^	0.50		
Limestone	1.10		
Calcium phosphate dibasic	0.70		
Salt	0.40		
Sodium bicarbonate	0.80		
Total	100.00		

a The premix provided the following nutrient content for one kilogram of diet: vitamin A, 6000 IU; vitamin D3, 2,500 IU; vita-min E, 12.5 IU; vitamin K3, 31.8 mg; vitamin B1, 0.035; vitamin B2, 8.5 mg; vitamin B6, 0.9 mg; nicotinic acid, 22 mg; D-pantothenic acid, 17 mg; vitamin B12, 0.03 mg; biotin, 0.14 mg, folic acid, 1.5 mg; Fe, 0.04 g; Cu, 0.008 g; Zn,0.05 g; Mn, 0.03 g; I,0.3 mg, Se,0.3 mg; Co, 0.25 mg.

b Digestible energy was calculated according to the Nutritional Requirements of Meat Sheep (NY/T 816-2021), while the rest were measured values.

### 2.2 Preparation of WEAA

The extract utilized in this research originated from *A. annua* gathered in Hohhot, Inner Mongolia. The WEAA was produced according to the protocol detailed by Guo et al. (25). Briefly, the *A. annua* were shade-dried, cut into small pieces, and then processed through decoction, concentration, and finally, freeze-drying. The extraction process involved incubating the plant material in water (1:25 ratio) at 80°C for 7 h. Subsequently, the mixture underwent vacuum filtration, and the filtrate was concentrated at a temperature of 80°C by a rotary evaporator. The concentrated solution was then freeze-dried using an ALPHA1-2LD plus freeze-drying machine (Christ, Germany) until a powdered extract was obtained. All extracts were stored at 4°C for future use. We used UPLC-Orbitrap-MS system to identify the structures of the active ingredients of WEAA, according to the method used by Xin et al. (26). The results are shown in Table 2.

**Table 2:** Compound contents of WEAA (DM basis, %).

Compounds	Contents
Organic acids and derivatives	24.61
Soluble polysaccharide	18.64
Flavonoids	9.80
Prenol lipids	7.75
Organoheterocyclic compounds	7.75
Organooxygen compounds	5.01
Nucleosides, nucleotides, and analogues	5.01
Fatty acyls	4.79
Benzene and substituted derivatives	3.87
Glycerophospholipids	2.28
Coumarins and derivatives	2.05
Cinnamic acids and derivatives	1.82
Phenols	1.60
Others	5.01

### 2.3 Sample collection and preparation of intestinal homogenate

On day 60 of the experiment, all 32 lambs (4 groups × 8 replicates, 1 lamb per replicate) were fasted for 12 h and then slaughtered at a commercial slaughterhouse by professionals, following the National Standard Operating Procedures (GB/T 43562-2023, *Code of Practice for Livestock and Poultry Slaughtering Operation—Sheep*, China). Each lamb was rendered unconscious using an electrical stunning method, exsanguinated via the jugular vein to induce death, and subsequently had their intestinal tissue collected. Samples from the duodenum, jejunum, and ileum were finely minced and homogenized using a hand-held homogenizer was employed in a 0.9% sodium chloride solution, kept ice-cold at 4°C, with a mixture ratio of 10% (w/v). After homogenization, the mixture was centrifuged at 3000 rpm for a duration of 10 min at 4°C. The supernatant obtained was then gathered for further analysis (27).

### 2.4 Analyses of intestinal immune and antioxidative indicators

Immune and antioxidant markers were evaluated through commercially available assays. Levels of IgA, IgG, IgM, IL-1β, IL-2, IL-4, IL-6, and TNF-*α* in the duodenum, jejunum, and ileum were determined with ELISA kits (Wuhan Colorful Gene Biological Technology Co., Ltd., Wuhan, China; 96 T per kit each), following the instructions set forth by the manufacturer. Additionally, antioxidant indicators (SOD, CAT, GSH-Px, MDA and T-AOC) were assessed with a V-1000 spectrophotometer (AOE Instruments Shanghai Co., Ltd., Shanghai, China) in the duodenum, jejunum, and ileum. These measurements kits were purchased from Jiancheng Bioengineering Institute, located in Nanjing, China. All procedures were performed in accordance with the manufacturer’s guidelines, and samples were assayed in duplicate.

### 2.5 Reverse transcriptase-quantitative PCR (RT-qPCR)

#### 2.5.1 RNA extraction and cDNA synthesis

Total RNA was isolated from small intestine tissues (duodenum, jejunum, ileum) using TRIzol reagent (TaKaRa Biotechnology, Dalian, China). RNA concentration was quantified using a Pultton P200 + spectrophotometer (Plextech, San Jose, CA, United States), and purity was assessed by A₂₆₀/A₂₈₀ ratio (1.8–2.1). RNA integrity was verified via 1% agarose gel electrophoresis. Genomic DNA contamination was eliminated by DNase I treatment (TaKaRa). cDNA was synthesized using the PrimeScript RT Master Mix kit (TaKaRa) on a LifeECO thermal cycler (Bori Technology, Hangzhou, China), following the protocol: 37°C for 15 min, 85°C for 5 s.

#### 2.5.2 Quantitative real-time PCR (qRT-PCR)

The target genes and their specific primer sequences can be found in Table 3. Quantitative real-time PCR (qRT-PCR) was performed using the LightCycler^®^ 96 System (Roche Diagnostics, Switzerland) and the TB^®^ Premix Ex Taq^™^ Kit (TaKaRa). qPCR Cycling Conditions: pre—denaturation at 95°C for 30 s; followed by 45 cycles of denaturation at 95°C for 5 s; annealing at 60°C for 30 s; extension at 72°C for 20 s; followed by melting curve analysis (95°C for 15 s, 60°C for 1 min, 95°C for 15 s). All reactions were performed in strict accordance with the manufacturers’ protocols (28).

**Table 3:** Primer sequences and parameter.

Gene	Sequence (5′- > 3′)^(1)^	GenBank no.	Length/bp
β-actin	F- ACAATGTGGCCGAGGACTTT	NM_001009784.3	278
	R- GCCGTGATGGCTGACCATTC		
GAPDH	F- TTATGACCACTGTCCACGCC	NM_001190390.1	216
	R- TCAGATCCACAACGGACACG		
Nrf2	F- TGTGGAGGAGTTCAACGAGC	XM_004004557.1	88
	R- CGCCGCCATCTTGTTCTTG		
Keap1	F- TTCAACAGCGAAAGTCAGGC	XM_027969637.2	157
	R- TGCGTAGCCTCCGATACTCT		
SOD1	F- GGAGACCTGGGCAATGTGAA	NM_001145185	182
	R- CCTCCAGCGTTTCCAGTCTT		
SOD2	F- AAACCGTCAGCCTTACACC	NM_001280703.1	116
	R- ACAAGCCACGCTCAGAAAC		
CAT	F- GAGCCCACCTGCAAAGTTCT	XM_004016396	148
	R- CTCCTACTGGATTACCGGCG		
GSH-Px	F- TGGTCGTACTCGGCTTCCC	XM_004018462.1	163
	R- AGCGGATGCGCCTTCTCG		
HO-1	F- CGATGGGTCCTCACACTCAG	XM_027967703.2	74
	R- CACACTCGCATTCACATGGC		
NQO1	F- CTCTGGCCAATTCAGAGTGG	XM_004015102.5	296
	R- TCCATTGGGATGGACTTGCC		
TLR4	F- CCTTGCGTACAGGTTGTTCC	NM_001135930.1	99
	R- GTCCAGCATCTCGGTTGACA		
MyD88	F- ATTGAGAAGAGGTGCCGTCG	NM_001166183.1	189
	R- ACAGACAGTGATGAAGCGCA		
IKKβ	F- GCCGCCCATTACAAGCTGAA	XM_042241396.1	165
	R- CTGGAAGAACGGGAGGTTCC		
IκB-α	F- TCACCTACCAGGGCTACTCC	NM_001166184.1	153
	R- CTGTGAACTCTGATTCGGTGTC		
NF-κBp50	F- GATGCCACTGCCAACAGAGA	XM_042251202.1	191
	R- GCGTCTGTCATTCGTGCTTC		
NF-κBp65	F- CTCCTCTCGGGGGATGAAGA	XM_027959295.2	123
	R- ATCCCTTGCTAACCCACTGC		
IL-1β	F- CTGTGGCCTTGGGTATCAGG	NM_001009465.2	251
	R- GCCACCTCTAAAACGTCCCA		
IL-4	F- GCTGAACATCCTCACATCGAG	AF1721681	87
	R- TTCTCAGTTGCGTTCTTTGG		
TNF-α	F- AGTCTGGGCAGGTCTACTTTG	NM_001024860	127
	R- GGTAACTGAGGTGGGAGAGG		
iNOS	F- AGACTGAGCCTCTCTAGCCC	XM_042255454.1	96
	R- GGAACCGTCTATAGCTGCCC		
COX-2	F- ACTTTCACGACCACACATTA	NC_001941.1	501
	R- GACGAGTTGACATAAGGGTT		

^(1)^ F: forward primer; R: reverse primer.

#### 2.5.3 Data analysis

Melting curves were analyzed to verify single peaks for product specificity. Outliers in triplicate Ct values (*n* = 3) were excluded, and the relative fold difference in mRNA expression levels was calculated using the 2^-∆∆Ct^ method (58).

### 2.6 Statistical analysis

All data were first processed using Microsoft Excel 2021 and then subjected to quadratic polynomial regression analysis with SAS Version 9.2 (2008, SAS Institute, Cary, NC, United States). Specifically, the analysis evaluated both linear and quadratic effects of dietary WEAA levels on various indices, in order to determine of dose-dependent relationships. Results were reported as mean and standard error of the mean (SEM), with *p* < 0.05 as the threshold for statistical significance. Additionally, a *p*-value within the range of 0.05 ≤ *p* < 0.10 was considered as a tendency.

## 3 Result

### 3.1 Small intestine inflammatory cytokines and immunoglobulins

The influence of WEAA supplementation on immunoglobulin levels in the small intestine of lambs is shown in Table 4. With increasing WEAA supplementation, the duodenal sIgA level demonstrated a linear upward trend (*p* < 0.10) and a significant quadratic increase (*p* < 0.05), whereas the IgG level exhibited a linear or quadratic increase (*p* < 0.05). The jejunal sIgA level demonstrated a quadratic increasing trend (*p* < 0.10), while IgG levels exhibited a linear upward trend (*p* < 0.10) and a significant quadratic increase (*p* < 0.05), peaking at 1000 mg/kg WEAA group. Additionally, WEAA supplementation did not significantly affect immunoglobulin levels in ileum tissue (*p* > 0.10).

**Table 4:** Effects of WEAA on immunoglobulins in the small intestine of lambs.

Item	WEAA mg/kg				SEM	*P*-value	
	0	500	1,000	1,500		Linear	Quadratic
sIgA, μg/mg prot.							
Duodenum	2.78	3.10	4.00	3.13	0.22	0.068	0.004
Jejunum	2.24	2.62	2.77	2.54	0.29	0.183	0.080
Ileum	3.04	2.95	3.20	3.42	0.10	0.099	0.181
IgG, μg/mg prot.							
Duodenum	5.78	7.23	8.75	7.19	0.32	0.045	0.005
Jejunum	4.96	6.15	6.86	5.91	0.41	0.056	0.003
Ileum	5.79	5.54	6.13	6.18	0.15	0.180	0.353
IgM, μg/mg prot.							
Duodenum	6.05	6.69	7.45	6.42	0.23	0.367	0.116
Jejunum	4.58	4.42	4.59	4.33	0.38	0.726	0.932
Ileum	4.84	4.96	5.55	5.08	0.20	0.477	0.603

In Table 5, intestinal inflammatory cytokine contents were shown. With increasing WEAA supplementation, IL-1β content of duodenum tissue demonstrated a significant linear increase (*p* < 0.05) and a quadratic trend (*p* < 0.10), while jejunal IL-1β level exhibited a quadratic upward trend (*p* < 0.10). Additionally, the duodenal IL-2 and IL-4 levels exhibited a significant quadratic increase (*p* < 0.05), and the jejunal and ileal IL-2 levels showed a linear or quadratic increase (*p* < 0.05), while jejunal IL-4 level exhibited a significant linear or quadratic increase (*p* < 0.05). Meanwhile, the ileal IL-4 content tended to increase linearly (*p* < 0.10).

**Table 5:** Effects of WEAA on inflammatory cytokines in the small intestine of lambs.

Item	WEAA mg/kg				SEM	*P*-value	
	0	500	1,000	1,500		Linear	Quadratic
IL-1β, pg./mg prot.							
Duodenum	68.99	72.30	79.67	78.02	3.83	0.032	0.082
Jejunum	74.95	81.76	88.35	80.47	6.95	0.188	0.065
Ileum	86.33	83.08	85.09	89.03	3.09	0.598	0.612
IL-2, pg./mg prot.							
Duodenum	60.64	67.82	82.61	61.77	2.50	0.425	0.010
Jejunum	47.41	58.01	57.34	58.05	4.74	0.043	0.043
Ileum	48.52	49.83	62.60	65.77	2.56	0.003	0.014
IL-4, pg./mg prot.							
Duodenum	23.03	30.39	34.28	26.35	1.11	0.169	<0.001
Jejunum	21.79	33.13	35.26	32.98	2.18	<0.001	<0.001
Ileum	25.37	23.28	29.71	29.15	1.09	0.067	0.181
IL-6, pg./mg prot.							
Duodenum	34.09	33.42	34.25	31.95	0.96	0.349	0.540
Jejunum	33.70	32.20	31.25	29.59	1.96	0.126	0.315
Ileum	30.48	20.63	27.23	25.34	1.26	0.441	0.215
TNF-α, pg./mg prot.							
Duodenum	69.52	70.53	70.67	70.76	2.68	0.801	0.961
Jejunum	73.99	74.15	70.98	73.29	6.06	0.781	0.932
Ileum	79.07	77.38	81.15	81.03	2.28	0.645	0.889

### 3.2 Small intestine antioxidative indexes

The effects of WEAA on the antioxidative indexes in the intestinal tissue of lambs is showed in Table 6. With increased WEAA supplementation, the jejunal T-AOC demonstrated a significant quadratic increase (*p* < 0.05), while the ileal T-AOC exhibited a linear increasing trend (*p* < 0.10). The duodenal GSH-Px concentration demonstrated a significant quadratic increase (*p* < 0.05), and jejunal GSH-Px concentration tended to increase quadratically (*p* < 0.10). Moreover, with the increase of WEAA dose, the ileal GSH-Px, duodenal and jejunal CAT concentration revealed a significant linear or quadratic increase effect (*p* < 0.05), while the duodenal and jejunal MDA content demonstrated a linear or quadratic decrease effect (*p* < 0.05), in addition, the concentration of MDA in the ileal tended to decline linearly (*p* < 0.10).

**Table 6:** Effects of WEAA on antioxidative indexes in the small intestine of lambs.

Item	WEAA mg/kg				SEM	*P*-value	
	0	500	1,000	1,500		Linear	Quadratic
T-AOC, μmol/mg prot.							
Duodenum	0.07	0.07	0.08	0.08	0.00	0.147	0.250
Jejunum	0.04	0.05	0.05	0.04	0.00	0.385	0.017
Ileum	0.04	0.04	0.05	0.05	0.00	0.062	0.136
T-SOD, U/mg prot.							
Duodenum	152.88	166.35	169.85	154.60	14.02	0.814	0.200
Jejunum	88.16	91.12	92.42	85.17	3.33	0.528	0.128
Ileum	93.30	96.04	98.11	98.63	2.63	0.214	0.443
GSH-Px, U/mg prot.							
Duodenum	48.30	58.44	72.72	49.87	3.38	0.381	<0.001
Jejunum	23.90	26.38	27.96	20.88	1.75	0.478	0.086
Ileum	20.16	25.21	32.12	35.47	1.72	<0.001	0.001
CAT, U/mg prot.							
Duodenum	12.36	22.12	22.28	19.01	1.62	0.025	<0.001
Jejunum	14.37	21.33	26.43	24.59	1.42	<0.001	<0.001
Ileum	13.52	16.80	13.21	17.69	0.86	0.252	0.494
MDA, nmol/mg prot.							
Duodenum	2.78	2.64	2.45	1.80	0.19	0.010	0.023
Jejunum	1.35	1.02	1.00	1.11	0.14	0.017	<0.001
Ileum	0.78	0.65	0.61	0.64	0.03	0.090	0.108

### 3.3 Duodenum TLR4/NF-κB and Nrf2/Keap1 pathway related gene expression

**Figure 1:**
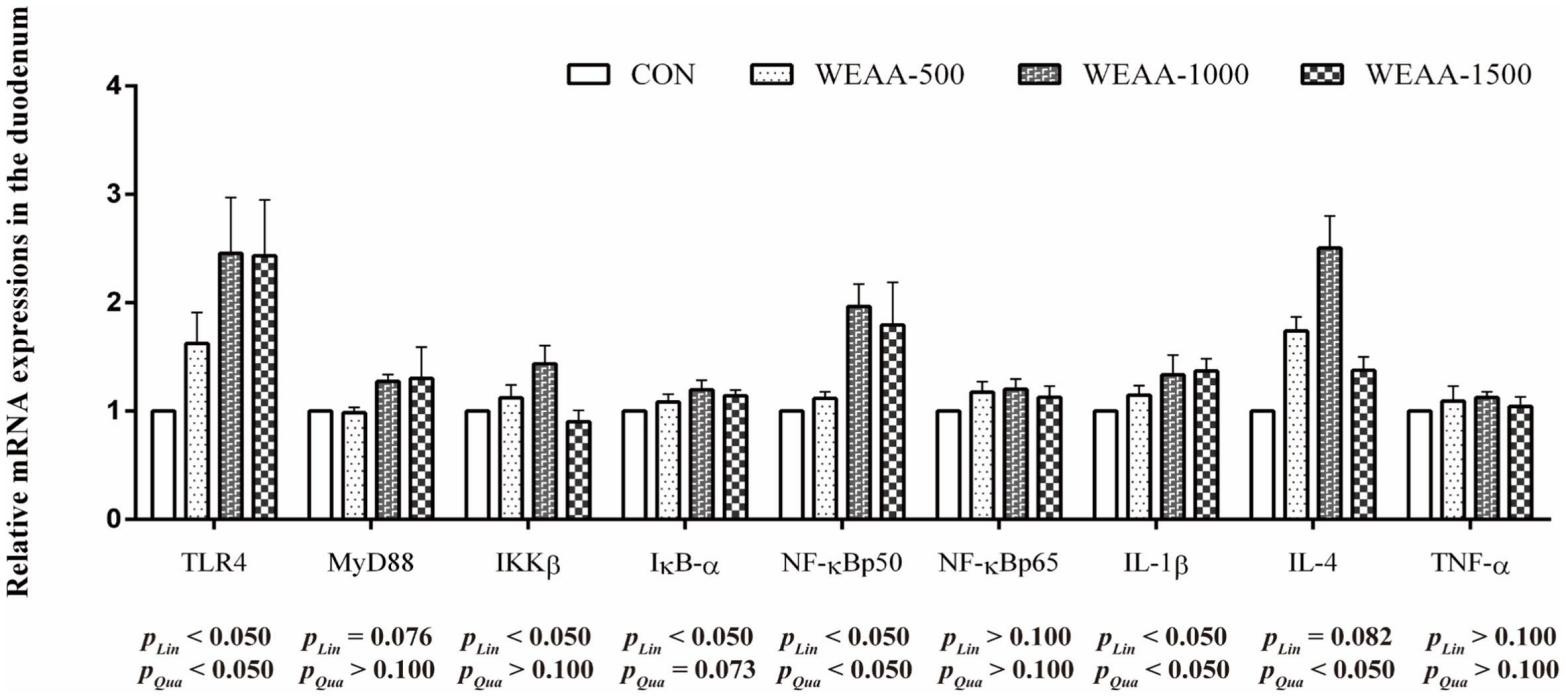
Effects of WEAA on expression of immune-related genes in the duodenum of lambs. Dose-dependent effects of WEAA (Lin: Linear; Qua: Quadratic) and the same as below.

Figure 1 shows the levels of mRNA expressions of immune-related genes in the duodenum. With the supplementation of WEAA increased, the expression levels of *TLR4*, *NF-κBp50* and *IL-1β* demonstrated a significant linear or quadratic increase effect (*p* < 0.05). The expression levels of *MyD88* exhibited a linear upward trend (*p* < 0.10). The expression levels of *IKKβ* demonstrated a linear increase (*p* < 0.05). Meanwhile, the expression levels of *IκB-α* exhibited a linear increase (*p* < 0.05) and a quadratic increasing trend (*p* < 0.10). In addition, the *IL-4* expression exhibited a linear increasing trend (*p* < 0.10), and a significant quadratic increase (*p* < 0.05).

**Figure 2:**
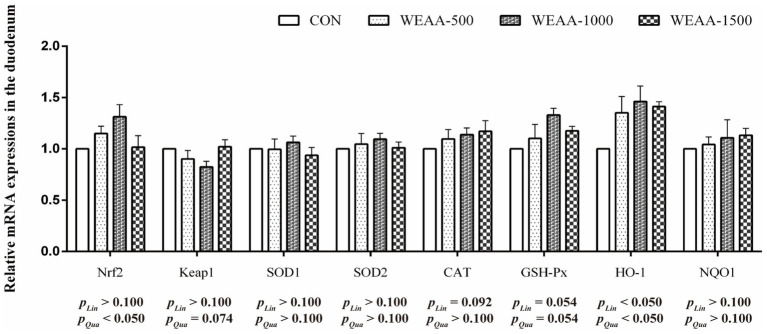
Effects of WEAA on expression of antioxidant-related genes in the duodenum of lambs.

Figure 2 showed the levels of mRNA expression related to antioxidant genes within the duodenum. With the supplementation of WEAA increased, the expression levels of *Nrf2* revealed an effect of increasing quadratically (*p* < 0.05). The expression levels of *Keap1* demonstrated a significant quadratic downward trend (*p* < 0.10), while the expression levels of *CAT* and *GSH-Px* revealed a linear upward trend (*p* < 0.10) and *GSH-Px* gene expression revealed a quadratic increasing trend (*p* < 0.10). Moreover, with the supplementation of WEAA increased, the expression level of *HO-1* revealed a significant linear or quadratic increase (*p* < 0.05).

### 3.4 Jejunum TLR4/NF-κB and Nrf2/Keap1 pathway related gene expression

**Figure 3:**
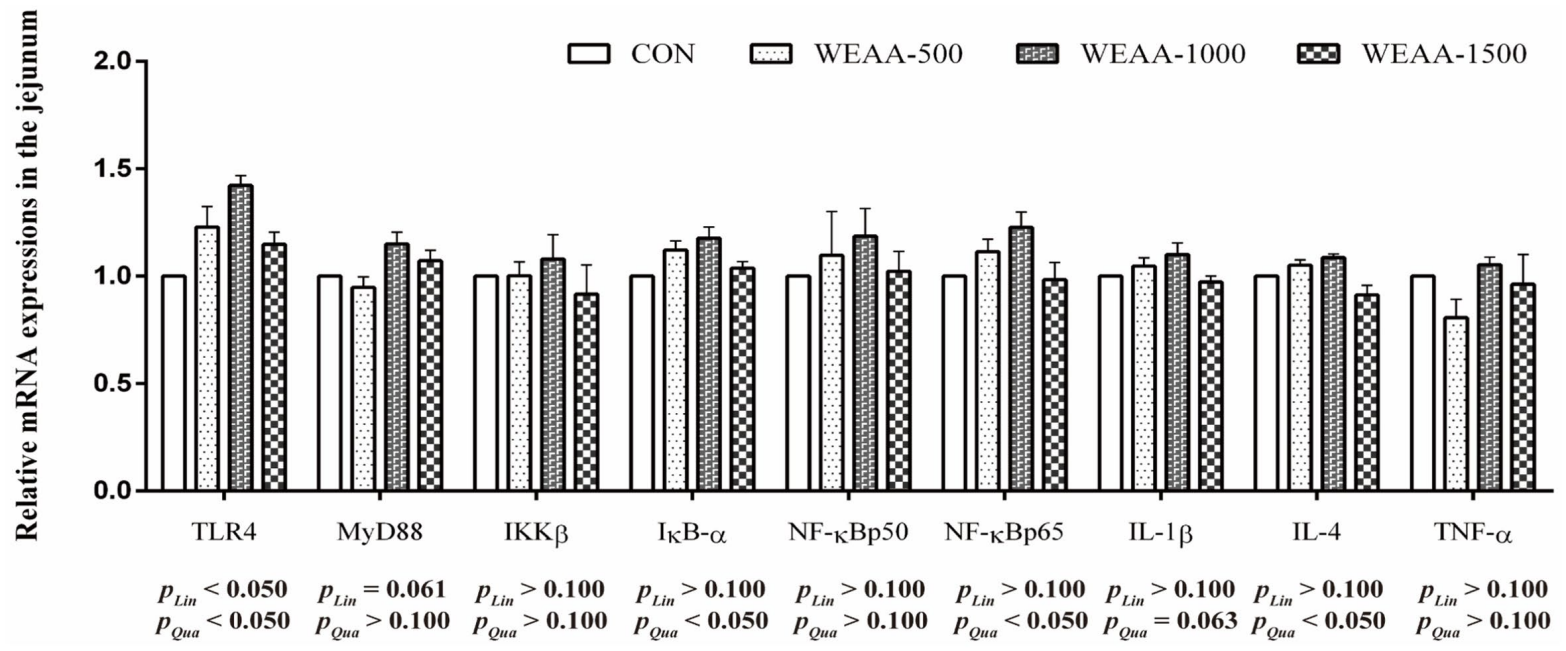
Effects of WEAA on expression of immune-related genes in the jejunum of lambs.

Figure 3 revealed the relative mRNA expressions levels of immune-related genes in the jejunum. With the supplementation of WEAA increased, the expression of *TLR4* demonstrated a significant linear or quadratic increase (*p* < 0.05). The expression levels of *MyD88* demonstrated a linear increasing trend (*p* < 0.10). Meanwhile, the expression of *IκB-α*, *NF-κBp65* and *IL-4* demonstrated a significant quadratic increase (*p* < 0.05), and the expression of *IL-1β* revealed a quadratic upward trend (*p* < 0.10).

**Figure 4:**
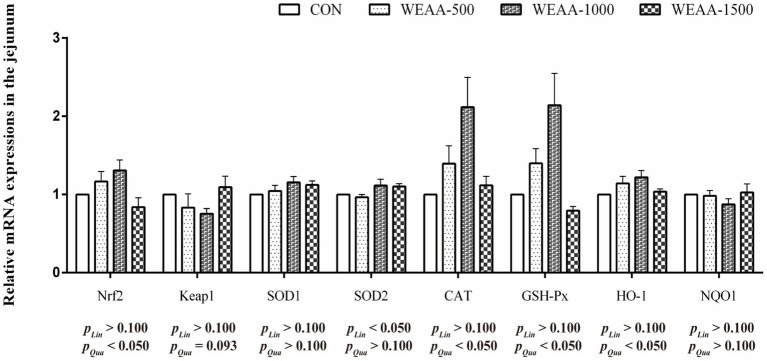
Effects of WEAA on expression of antioxidant-related genes in the jejunum of lambs.

Figure 4 showed the relative mRNA expressions of relative antioxidant genes in the jejunum. With the supplementation of WEAA increased, *Nrf2*, *CAT*, *GSH-Px* and *HO-1* demonstrated a significant quadratic increase (*p* < 0.05). The expression levels of *Keap1* revealed a quadratic downward trend (*p* < 0.10). Meanwhile, the expression of *SOD2* demonstrated a significant linear increase effect (*p* < 0.05).

### 3.5 Ileum TLR4/NF-κB and Nrf2/Keap1 pathway related gene expression

**Figure 5:**
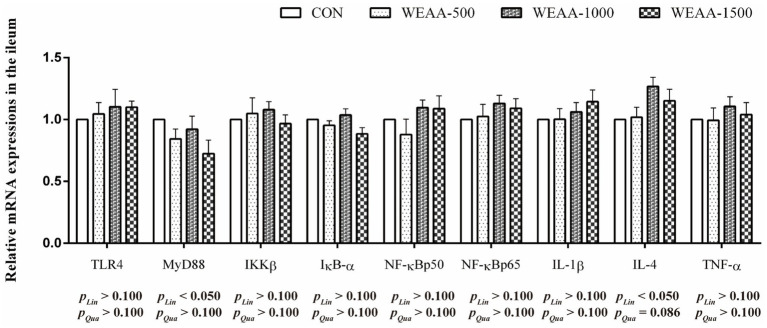
Effects of WEAA on expression of immune-related genes in the ileum of lambs.

Figure 5 demonstrated the relative expressions of immune-related genes in the ileum. With the supplementation of WEAA increased, the expression of *MyD88* and *IL-4* revealed a significant linear increase (*p* < 0.05). Meanwhile, the *IL-4* gene expression demonstrated a quadratic increasing trend (*p* < 0.10).

**Figure 6:**
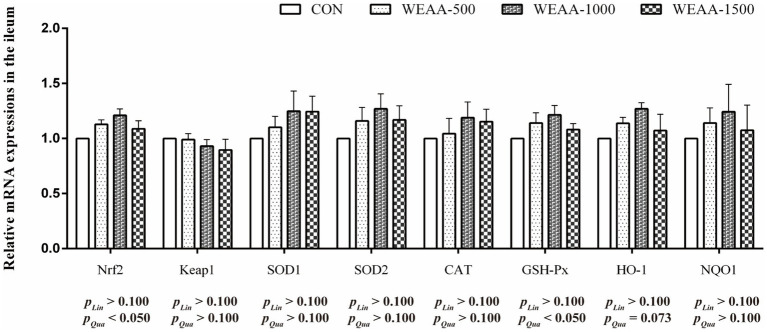
Effects of WEAA on expression of antioxidant-related genes in the ileum of lambs.

Figure 6 showed the relative expressions levels of relative antioxidant genes in the ileum. With the supplementation of WEAA increased, the expression levels of *Nrf2* and *GSH-Px* revealed a significant quadratic increase (*p* < 0.05). Meanwhile, the *HO-1* expression demonstrated a quadratic upward trend (*p* < 0.10).

## 4 Discussion

*A. annua* is a traditional medicinal plant from China, well-known for its significant biological effectiveness and minimal toxicity. It is typically prepared as decoctions to break down the plant cell wall, thus aiding in the release of bioactive constituents (29). Notably, among these constituents are polysaccharides, flavonoids, and sesquiterpenes, which have particular relevance in the field of immunopharmacology (22) *A. annua* contains a variety of active components, such as flavonoids, polysaccharides, and sesquiterpenes (17, 30). In the present study, the identification of active ingredients by structure analysis for WEAA indicated the presence of organic acids, polysaccharide, flavonoids and prenol lipids, which were the dominant substances (Table 2). Previous research indicated that flavonoids from *Artemisia ordosica* that significantly boost antioxidant enzyme activity when added to broiler diets (31). Moreover, extracts from *A. annua* have shown antioxidative properties in mouse models (32). Additionally, polysaccharides obtained from the leaves of *Artemisia argyi* have been found to possess antioxidant and immune-boosting abilities (33, 34).

This study observed elevated sIgA and IgG concentrations in the duodenum and jejunum of lambs as dietary intake of WEAA increased. This result agrees with the findings of Guo et al. (35), who demonstrated that a diet high in WEAA increased the concentrations of sIgA, IgG, and IgM in the small intestine of broiler chickens. Immunoglobulins, which are antigen-specific receptors, are essential for humoral immunity as they bind to specific antigens, such as flavonoids and plant polysaccharides, thereby triggering immune cell signaling that initiates effective adaptive immune responses (36). Specifically, sIgA is essential for the maintenance of intestinal homeostasis, that is, maintaining the integrity of biological barriers and limiting microorganism and antigen exposure. Moreover, the immune response is directly influenced by IgG, which is produced by B cells (37). Supporting these findings, Yang et al. (38) demonstrated that the inclusion of alcohol extracts from *Artemisia argyi* in the diet mitigated the adverse effects induced by LPS challenges. This effect is attributed to an increase in immunoglobulin levels (IgA, IgG, and IgM) in the small intestine of broiler chickens, suggesting an enhanced immune response in the intestinal tract. Moreover, Zhang et al. (39) investigated impact of water extracts from *Artemisia rupestris* L. (AEAR) in both *in vivo* and *in vitro*, showing that AEAR significantly increased IgG titers in ICR mice, partly by promoting dendritic cell maturation via the TLR4 signaling pathway, thereby facilitating immune cell proliferation, differentiation, and autoantibody production.

TNF-*α*, IL-1β, and IL-6 are crucial inflammatory cytokines in the intestinal immune system, where they activate adaptive immune responses to combat pathogenic bacterial invasion. However, their excessive expression can damage mucosal cells. Song et al. (23) demonstrated that exposure to heat enhanced the *TLR4* and pro-inflammatory cytokines expression in the intestinal of broiler chickens. However, pretreatment with EA mitigated the increase of IL-1β expression levels of *TLR4* and *IL-6* in the intestinal regions under heat stress. Additionally, a dietary supplement of 0.1% *A. annua* extract led to a decrease in both the content and expression of inflammatory cytokines within the small intestine of both neonatal and post-weaning piglets (15). These results suggest that *A. annua* plays a beneficial role in alleviating intestinal inflammation. Yang et al. (40) also demonstrated that AEAR stimulated the content of IL-1β and IL-6 through the activation of TLR2/4 signaling pathways in dendritic cells, without inducing toxicity. Du et al. (41) demonstrated that polysaccharides derived from *Artemisia ordosica* significantly stimulated inflammatory cytokine production in the small intestine with a dose-dependent manner, thereby improved the immune function of broilers under conventional feeding conditions. Similarly, *Artemisia argyi* polysaccharide at an optimal dosage regulated immune functions, elevating cytokine levels (IL-1β, IL-2, and IL-4) and IFN-*γ* in broiler serum (42). The present study suggested that supplementing the diet with WEAA might enhance the content and gene expression of IL-1β, IL-2, and IL-4 in the duodenum and jejunum of lambs. These results suggested that WEAA could play a significant role in modulating immune responses.

In order to delve deeper into the molecular mechanisms underlying the impact of WEAA addition on intestinal immunoregulation, we detected the expression levels of crucial mRNAs associated with the TLR4/NF-κB signaling cascades. *A. annua*, a well-known medicinal plant, primarily comprises polysaccharides, flavonoids, and artemisinin as its main components which are noted for their structural diversity and enhance their affinity with various pattern recognition receptors (PRRs), thereby initiating signal pathways related to immune response. TLR4, a pattern recognition receptor belonging to the TLR family. This receptor is essential for immune responses and is present in nearly all types of intestinal epithelial cells. Research identified that water-soluble polysaccharide from *Artemisia rupestris* L. interacted with TLR2/4 on dendritic cells, facilitating cytokine production and signal transduction processes. This interaction not only enhanced the phosphorylation levels of IKKβ and NF-κBp65 but also stimulated the degradation of IκB-*α* in the cytoplasm and promoted the translocation of NF-κBp65 into the nucleus (39). These mechanism insights suggest that the elicitation of NF-κB via TLR2/4 pathways by these polysaccharides is crucial for cytokine synthesis. Further studies by Ando et al. (43) uncovered that safflower polysaccharides could activate TLR4/NF-κB pathway, thereby inducing cytokine production in macrophages. Additionally, Guo et al. (44) found that *A. annua* polysaccharide alleviated excessive release of intestinal pro-inflammatory factors induced by *E. coli* challenge in broilers via inhibiting TLR4/MyD88/NF-κB signaling pathway, and up-regulated anti-inflammatory factor IL-4 and immunoglobulins (IgA, IgG, IgM) to synergistically enhance immune regulation; moreover, *A. annua* polysaccharide also increased serum IgA in non-challenged broilers, reversed *E. coli*-induced decrease in serum IgM and jejunal IgG/IgM, and promoted IL-4 secretion, furthermore, enhanced anti-inflammatory effect. These results suggest that *A. annua* polysaccharide coordinates immune regulation by modulating TLR4/NF-κB pathway and immune effector molecules. The present study found that dietary supplementation with WEAA could significantly enhance *TLR4* expression in the duodenum and jejunum of lambs, and up-regulated the gene expression levels of its downstream signaling molecules *MyD88*, *IKKβ*, *IκBα*, *NF-κBp50* and *NF-κBp65*. The results suggest that WEAA may recognize extracellular signals through activating TLR4 receptors, thereby initiating the cascade activation of the TLR4/MyD88/NF-κB signaling pathway. It was noteworthy that the significant increase in *IκBα* expression might be due to the transcription and expression of *IκBα* induced by the pathway to form negative feedback regulation to limit the excessive activation of NF-κB (45), thereby avoiding excessive inflammatory response in intestinal tissues. Generally, the activation of the NF-κB signaling pathway promotes the pathway’s binding to the κB response element of target genes, and initiates the transcription of pro-inflammatory cytokines such as TNF-α, IL-1β, and IL-6, mediates inflammatory responses and immune responses, and indirectly promotes the production of immunoglobulins (46), regulates the secretion of T cell-related cytokines (IL-2), and affects the secretion of IL-4 by Th2 cells, thereby enhancing the body’s immune function from multiple dimensions (47). Some studies found that AEAR significantly increased the gene expression of IL-1β and IL-2 and the protein expression level of NF-κB in dendritic cells, which significantly enhanced the immunomodulatory activity of dendritic cells (40). Furthermore, polysaccharides derived from *Artemisia ordosica* were reported to enhance the contents of immunoglobulins (IgG, IgA, IgM) and cytokines (IL-1β, IL-2, IL-4), also to increase the mRNA expression of the *TLR4*, *MyD88*, *NF-κBp50*, and *IL-1β* in the small intestine of broilers (41). Similarly, the current study revealed that supplementing WEAA into the diet up-regulated the mRNA expression of *IL-1β* in the duodenum and jejunum of lambs, which was consistent with the increase in its content. Additionally, the trend of its change was similar to the expression of *NF-κB*. Meanwhile, the content of IL-4 and its mRNA expression level were also significantly increased, demonstrating the anti-inflammatory activity of WEAA. Based on the above results, it is speculated that under normal physiological conditions, WEAA can synergistically improve the immune regulation function of the body by regulating the dynamic balance of the TLR4/NF-κB signaling pathway. In addition, a relatively significant change in relative expressions of the related factors was noted in the small intestine that is located in its proximal and middle regions of lambs.

Reactive oxygen species (ROS) are produced throughout the normal growth and metabolic processes of mammals. They play a crucial and continuous role in several functions, including intracellular signal transduction and the modulation and activation of immune responses. If these radicals are not promptly scavenged, they accumulate in the body, damaging cellular morphology and function. The peroxidation of unsaturated lipids within biological membranes results in the formation of lipid peroxides and malondialdehyde, which precipitate a cascade of inflammatory changes (48). The antioxidant defense system has a variety of endogenous antioxidant enzymes in cells, which are the first line of defense against oxidative stress. Specifically, Mn-SOD catalyzes the conversion of O^2−^ to e H_2_O_2_ and plays a significant role in activating associated signaling pathways that promote the activity of SOD1, CAT, GSH-Px, HO-1, thereby facilitating the detoxification of H_2_O_2_ into H_2_O (49). In the current research, dietary WEAA was found to improve the activity of CAT and GSH-Px in the duodenum. Meanwhile, it increased the activity of T-AOC, CAT, and GSH-Px in the jejunum, and the activity of GSH-Px in the ileum of lambs. This finding aligned with a prior study by Zhang et al. (50) on broilers, which stated that dietary *Artemisia argyi* powder could enhance intestinal antioxidative enzyme activities. Moreover, this study found that an increase in the relative gene expressions of CAT and GSH-Px were observed in the small intestine, and the changes in relative gene expression were consistent with the change in enzymatic activity. These results suggest that WEAA may improve small intestinal antioxidant capacity by regulating gene expression and activity of the antioxidant enzymes. By contrast, MDA is a reliable biomarker for the assessment of oxidative stress (51). The current study demonstrated that the dietary supplementation of WEAA to lambs MDA levels were found to decrease in duodenum and jejunum of lambs. The observation of Wan et al. (8) that *A. annua* leaves and EA can enhance antioxidant enzyme activities, while simultaneously reducing MDA levels in both the serum and liver of broilers support our research findings. This finding underscores the potential for *in vivo* antioxidant defense. Furthermore, Song et al. (23) reported that *A. annua* was rich in polysaccharides, phenolic and flavonoids. Their researches demonstrated that dietary addition with EA could alleviate the elevation of MDA concentration in the intestine induced by heat stress and relieve the stress response in broilers following such stress. As we all know, under the normal physiological conditions or the status with low levels of lipid peroxidation, organisms have an inherent capacity to regulate cellular metabolism. This regulatory mechanism via the activation of related signaling pathways to effectively enhance the activity of antioxidant enzymes. Consequently, this leads to an adaptive stress response that helps maintain cellular homeostasis (52). Nrf2 is a key regulatory factor with intracellular antioxidant and anti-inflammatory damage. Nrf2 dissociates from Keap1 into the nucleus and binds to antioxidant response elements (ARE), promoting the transcription of cell protective factors and upregulating the expression of related antioxidant genes. This leads to an increase in intracellular antioxidant enzyme levels, thereby clearing free radicals and reactive oxygen species, and resistance to oxidative damage (53). In addition, the activation of the Nrf2-ARE signaling pathway not only has the effect of anti-oxidative stress, but also enhance the body’s immune response (54). Therefore, the Nrf2/Keap1 signaling pathway, as a key protective pathway, has important biological significance for the immune and antioxidant functions of the body. Niu et al. (55) when they fed weaned piglets with EA, it could improve the intestinal antioxidant status. Specifically, the mRNA expression of *Nrf2* and *HO-1* was raised. Xing et al. (46) further suggested that the effect of adding polysaccharides derived from *Artemisia ordosica* to diet on lipopolysaccharide induced oxidative stress in broilers, and found can alleviate oxidative stress in broilers by regulating the Nrf2 signaling pathway and increasing the production of antioxidant enzymes. The current research demonstrated that WEAA greatly enhanced *Nrf2* and *HO-1* expression levels in intestine, accompanied by a significant decrease in *Keap1* gene expression. These findings showed that WEAA might enhance the antioxidant activity of intestinal tissue by activating Nrf2-mediated pathway related mRNA expressions. Therefore, WEAA emerges as a promising green feed additive capable of enhancing immunomodulatory and antioxidant capabilities within the intestines of lambs.

## 5 Conclusion

To summarize, this research showed that dietary supplementation of WEAA in lambs effectively modulated the TLR4/NF-κB signaling pathway in the duodenum and jejunum, promoting the production of immunoglobulins (sIgA, IgG) and cytokines (IL-1β, IL-2, IL-4). Concurrently, WEAA significantly upregulated Nrf2/HO-1 expression while downregulating Keap1, thereby activating the antioxidant pathway and enhancing antioxidant enzyme (CAT, GSH-Px) activity. The study determined that the optimal dosage of WEAA in lamb’s diet was 1,000 mg per kg of feed. Future studies should explore the molecular mechanisms for WEAA regulating TLR4/NF-κB and Keap1/Nrf2 pathways.

## Data Availability

The datasets presented in this study can be found in online repositories. The names of the repository/repositories and accession number(s) can be found in the article/supplementary material.
